# PET/MR attenuation correction in brain imaging using a continuous bone signal derived from UTE

**DOI:** 10.1186/2197-7364-2-S1-A39

**Published:** 2015-05-18

**Authors:** Claes Ladefoged, Didier Benoit, Ian Law, Soren Holm, Liselotte Hojgaard, Adam Espe Hansen, Flemming Littrup Andersen

**Affiliations:** Department of Clinical Physiology, Nuclear Medicine and PET, Rigshospitalet Copenhagen, Copenhagen, Denmark

In the absence of transmission sources in combined clinical PET/MR systems, MR images are used for MR-based attenuation correction (MRAC). The main challenge in MR-AC is to separate the bone and air, as neither have a signal in the MR images. In the attenuation maps supplied by the vendor, a single value is assigned to bone using an ultra-short echo time (UTE) MR sequence. The purpose of this study was to develop a new multi-class segmentation-based MR-AC method, employing Continuous-Bone-using-R2* (MRAC_CBuR2*), and evaluate it on a large patient cohort. 53 [18F]-FDG PET/MR brain patients were included in this study. MRAC was based on an aligned CT (MRAC_CT, used as reference), standard MRAC_UTE and MRAC_CBuR2*. Our method segments the air, brain, CSF and soft tissue voxels on the UTE images, and uses a mapping of R2* values to HU to measure the density in bone voxels. Aligned anatomical masks are used to improve accuracy in noisy regions. Region-based analysis was performed using ICBM 2009a brain atlas with anatomical labels pre-defined. Using CBuR2*, 82% of the voxels in the brain are within ±5% of PET_CT, compared to 27% when using UTE. Using our method, there are clear improvements over UTE. The average error over the full brain is 0.8% (±1.7%), compared to -7.1% (±2.4%) in UTE. Of note, the maximum error in the cerebellum is -15% and 7% in UTE and CBuR2*, respectively. The proposed method uses the available UTE images to segment tissue classes, and uses the R2* map to measure a continuous bone signal. The improvement over the vendor provided UTE reduces both the global and local error on the reconstructed PET images.

